# Jaceidin Flavonoid Isolated from *Chiliadenus* *montanus* Attenuates Tumor Progression in Mice via VEGF Inhibition: In Vivo and In Silico Studies

**DOI:** 10.3390/plants9081031

**Published:** 2020-08-14

**Authors:** Sameh S. Elhady, Enas E. Eltamany, Amera E. Shaaban, Alaa A. Bagalagel, Yosra A. Muhammad, Norhan M. El-Sayed, Seif N. Ayyad, Amal A. M. Ahmed, Mohamed S. Elgawish, Safwat A. Ahmed

**Affiliations:** 1 Department of Natural Products, Faculty of Pharmacy, King Abdulaziz University, Jeddah 21589, Saudi Arabia; ssahmed@kau.edu.sa; 2 Department of Pharmacognosy, Faculty of Pharmacy, Suez Canal University, 41522 Ismailia, Egypt; enastamany@gmail.com (E.E.E.); amera_ms247@yahoo.com (A.E.S.); 3 Department of Pharmacognosy, Faculty of Pharmacy, Horus University, New Damietta 34518, Egypt; 4 Department of Pharmacy Practice, Faculty of Pharmacy, King Abdulaziz University, Jeddah 21589, Saudi Arabia; abagalagel@kau.edu.sa; 5 Department of Pharmaceutical Chemistry, Faculty of Pharmacy, King Abdulaziz University, Jeddah 21589, Saudi Arabia; ymuhammad@kau.edu.sa; 6 Department of Pharmacology & Toxicology, Faculty of Pharmacy, Suez Canal University, 41522 Ismailia, Egypt; norhan.ms@pharm.suez.edu.eg; 7 Department of Organic Chemistry, Faculty of Science, Damietta University, New Damietta 34511, Egypt; snayyad2@yahoo.com; 8 Department of Cytology & Histology, Faculty of Veterinary Medicine, Suez Canal University, Ismailia 41522, Egypt; amal_ahmed122@yahoo.com; 9 Department of Medicinal Chemistry, Faculty of Pharmacy, Suez Canal University, Ismailia 41522, Egypt; mohamed_elgawish@pharm.suez.edu.eg

**Keywords:** *Chiliadenus montanus*, jaceidin, anti-tumor, Ehrlich’s ascites carcinoma, VEGF

## Abstract

Phytochemical study of *Chiliadenus montanus* aerial parts afforded six compounds; Intermedeol (1), 5α-hydroperoxy-β-eudesmol (2), 5,7-dihydroxy-3,3’,4’-trimethoxyflavone (3), 5,7,4’-trihydroxy-3,6,3’-trimethoxyflavone (jaceidin) (4), eudesm-11,13-ene-1β,4β,7α-triol (5) and 1β,4β,7β,11-tetrahydroxyeudesmane (6). These compounds were identified based on their NMR spectral data. The isolated compounds were tested for their cytotoxicity against liver cancer cell line (HepG2) and breast cancer cell line (MCF-7). Jaceidin flavonoid (4) exhibited the highest cytotoxic effect in vitro. Therefore, both of jaceidin and *C. montanus* extract were evaluated for their in vivo anti-tumor activity against Ehrlich’s ascites carcinoma (EAC). Compared to control group, jaceidin and *C. montanus* extract decreased the tumor weight, improved the histological picture of tumor cells, lowered the levels of VEGF and ameliorate the oxidative stress. Molecular docking and in silico studies suggested that jaceidin was a selective inhibitor of VEGF-mediated angiogenesis with excellent membrane permeability and oral bioavailability.

## 1. Introduction

Cancer is a non-communicable disease triggered by severe imbalance between cell proliferation and apoptosis which will lead to a massive expansion in neoplasms population [1]. Cancer is the second-leading cause of mortality and morbidity behind only heart disease [2]. Recent statistical reports estimated that, cancer has affected approximately 18.1 million people in 2018. In addition, up to 9.1 million cancer deaths were reported in 2018 and it is expected to reach 20.3 million in 2026. Most of deaths were correlated to breast cancer and hepatocellular carcinomas [3]. Cancer therapies have been found to be of low efficacy due to high toxicity, lack of selectivity and most importantly the ability of cancer cells to be resistant against them [3,4]. Therefore, several attempts have been made to develop novel therapeutic candidates to combat malignant tumors. Phytomedicines provide a new horizon for cancer therapy [3,5] for being safer and more available [4].

Newman and Cragg estimated that more than 50% of all modern clinical drugs were natural products [6]. Interestingly, about 49% of cancer drugs discovered from the 1940s to the end of 2014 were of natural sources [7] such as vinca alkaloids and paclitaxel. Moreover, etoposide (VP-16) and teniposide (VM-26) are derivatives of podophyllotoxin, isolated from mandrake plant (*Podophyllum peltatum*) [6,8]. *Chiliadenus montanus* (Vahl.) Brullo (= *Jasonia montana*, *Varthemia montana* (Vahl.) Boiss, *Chrysocoma montana* (Vahl.) Symb.), a medicinal plant belongs to family Asteraceae one of the largest angiosperm families. *C. montanus* locally known as haneida is indigenous to Sinai Peninsula in Egypt occurs in the rocky valleys and hillsides [9,10]. It has a good reputation among the traditional healers and Bedouins as a remedy for various ailments such as chest and kidney problems, diarrhea and stomachache [10,11,12]. Several biological studies have proved the hepatoprotective effect [13], the antidiabetic, antioxidant, anticholestatic and anti-obesity activities of the plant [14,15,16,17] and its ability to ameliorate the inflammation and neurodegeneration processes characterizing Alzheimer’s disease [18]. *C. montanus* owes its unique pharmacological activities to an array of secondary metabolites. Chemical investigation of *C. montanus* resulted in the isolation of different sesquiterpenoidal compounds mainly of eudesmane type [15,19,20,21,22]. Furthermore, numerous poly methoxylated and polyhydroxylated flavonoids have been reported [11,12,14,23] such as 5,7-dihydroxy-3,3’,4’-trimethoxyflavone and chrysosplenetin which have displayed chemoprotective and anticancer effects respectively [11,12].

Flavonoids exhibit multiple pharmacological activity including antiviral, anti-inflammatory, anti-aging, and protective effect against Alzheimer’s disease [24,25,26]. Moreover, plant flavonoids have been proven to be strong chemotherapeutic candidates against various types of cancer via modulating apoptotic pathway [27]. The major mechanism involves the activation of apoptotic proteins intrinsically and extrinsically, elevation of ROS, and induction of DNA damage in addition to their interference, with multiple signal transduction in the process of carcinogenesis and thus limit proliferation, angiogenesis and metastasis [1]. Therefore, researchers focused more interest to disclose the anticancer potential of flavonoids containing plants compounds due to their remarkable biological activities and devoted to overcome the limitations such as toxicity and poor solubility etc., [28]. Nowadays, the application of computer-based drug design has been progressively raised to investigate the biological activity of many natural compounds and in the development of new drug-like molecules [29]. Computer-based molecular docking simulations are a powerful strategy for predicting the coupling of medications with their receptor, which can be effectively executed to save on the effort, time, and resources required by traditional drug development methodology [30].

In continuation of efforts in discovering bioactive phytochemicals from Egyptian Traditional Medicine, four sesquiterpenes and two methoxyflavonoids were isolated from *C. montanus*. The in vitro cytotoxic activity of the isolated compounds was assessed. Then, the most active metabolite in vitro as well as *C. montanus* extract were investigated for their in vivo antitumor using Ehrlich’s ascites carcinoma mice model. Plasma vascular endothelial growth factor (VEGF) and some biomarkers of oxidative stress were assessed as possible mechanisms involved in explaining their anti-tumor effect. Furthermore, we inspected the interaction of the bioactive compound with active cavities of the target receptors by using molecular docking simulations.

## 2. Results and Discussion

### 2.1. Identification of the Isolated Compounds

**Figure 1:**
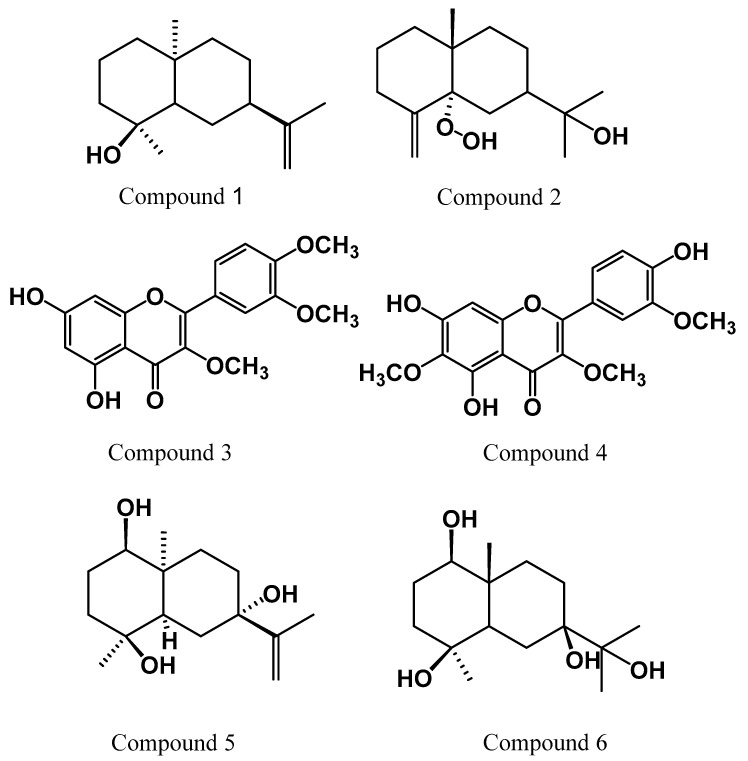
Chemical structures of the isolated compounds.

*C. montanus* afforded six compounds as shown in (Figure 1). Four of them were sesquiterpenoids (1, 2, 5 and 6) while compounds 3 and 4 were polymethoxylated flavonoids.

To the best of our knowledge, compounds 1, 2 and 6 are reported for the first time in genus *Chiliadenus* while compounds 3, 4 and 5 were previously isolated from this plant *C. montanus* [11,15,23].

### 2.2. Screening for Antitumor Activity

#### 2.2.1. In Vitro Assay

It is reported that sesquiterpenes and flavonoids from *C. montanus* exhibited potential cytotoxicity on human colon carcinoma (Caco-2) and human cervix cancer cells (HeLa) [12,19]. Hence, the isolated compounds were screened for their inhibitory effect on breast cancer cell line (MCF-7), liver cancer cell line (HepG2) and human melanocyte (normal cell line) (HFB-4) SRB assay with doxorubicin as positive control. The IC_50_ values are shown in Table 1. Our results showed that compound 4 (jaceidin) displayed remarkable cytotoxic activity on both MCF-7 and HepG2 with IC_50_ values 9.3 and 9.7 µM respectively. On the other hand, compound 3 5,7-dihydroxy-3,3’,4’-trimethoxyflavone exhibited less inhibition activity on HepG2 and MCF7 cancer cell lines with IC_50_ 30.5 and 10.7 µM respectively when compared to jaceidin, despite their structure similarity.

Results from the current study demonstrated that jaceidin was the most active metabolite isolated from *C. montanus* to have a prospective cytotoxic in vitro. As reported, excessive reactive oxygen species (ROS) leads to oxidative damage including cell lipids, DNA injury and then induces cell apoptosis [31]. Generally, flavonoids are direct scavengers of reactive oxygen species (ROS). However, the configuration and arrangement of functional groups greatly influence the efficacy of their ROS scavenging. Indeed, this was reflected on their cytotoxic effect in vitro. The B- ring hydroxyl configuration is the most significant determinant of strong radical scavenging of ROS and RNS and firmly enhances inhibition of lipid peroxidation because it donates hydrogen and electrons to hydroxyl, peroxyl and peroxynitrile radials [32].Therefore, this could explain why jaceidin showed a higher cytotoxic effect than 5,7-dihydroxy-3,3’,4’-trimethoxyflavone (compound 3). Secondly, the presence of 2,3-double bond in conjugation with a 4-oxo function in the C ring in both jaceidin and 5,7-dihydroxy-3,3’,4’-trimethoxyflavone (compound 3) contributed to their efficacy as cytotoxic agents. This could be attributed again to their ROS scavenging abilities as the presence of the 2,3 double bond in conjugation with a 4-keto function provides electron delocalization from the C ring [33]. After comparing the cytotoxicity of the isolated compounds as depicted in Table 1. Compound 4 had the highest inhibition effect against MCF7 and HepG2 cells. Therefore, jaceidin was selected for a detailed comparative study to assess the anti-tumor activity of jaceidin and the extract in vivo.

#### 2.2.2. In Vivo Assay

##### Tumor Weight

**Figure 2:**
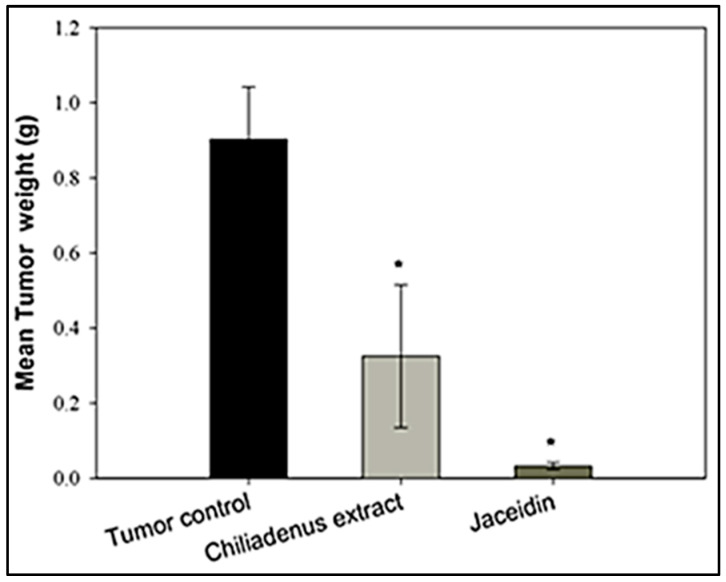
Effect of *Chiliadenus montanus* extract and Jaceidin on weight on Ehrlich’s ascites carcinoma solid tumors growing in female mice. Data are expressed as the mean ± SD and analyzed using one-way ANOVA followed by Bonferroni’s post-hoc test. * compared to the tumor control group at *p* < 0.05.

*Chiliadenus montanus* extract and jaceidin were evaluated for their antitumor activity in vivo using Ehrlich’s ascites carcinoma (EAC) solid tumors grown in female mice. *C. montanus* extract and jaceidin displayed highly significant antitumor activity in comparison to tumor control. The reduction in the tumor weight by jaceidin was greater than that produced by *C. montanus* extract in comparison with tumor control at *p* < 0.05 (Figure 2). The mean tumor weights of mice treated with *C. montanus* extract and mice treated with the jaceidin were 0.33 and 0.032 mg respectively compared to 0.9 mg in tumor control. The tumor weight in the *C. montanus* extract and jaceidin were decreased by 63.4% and 96.4% respectively compared to tumor control.

These findings are in line with those of in vitro cytotoxicity assay. In addition, they are in agreement with the in vivo antitumor activity of methoxylated flavonoids of *Achillea fragrantissima* [34] and *Gomphrena martiana* [35]. The in vivo efficacy of the methoxylated flavonoid may be attributed to their improved hepatic metabolic stability as they are less susceptible to glucuronic acid or sulfate conjugation. Moreover, methylation of flavonoids increases their intestinal absorption, and oral bioavailability [36,37]. In addition, methoxylated flavonoids are less toxic to vital cellular compounds containing SH-groups compared to their hydroxylated analogues as in case of tamarixetin and quercetin [38].

##### Histopathological Examination of Solid Tumors

**Figure 3:**
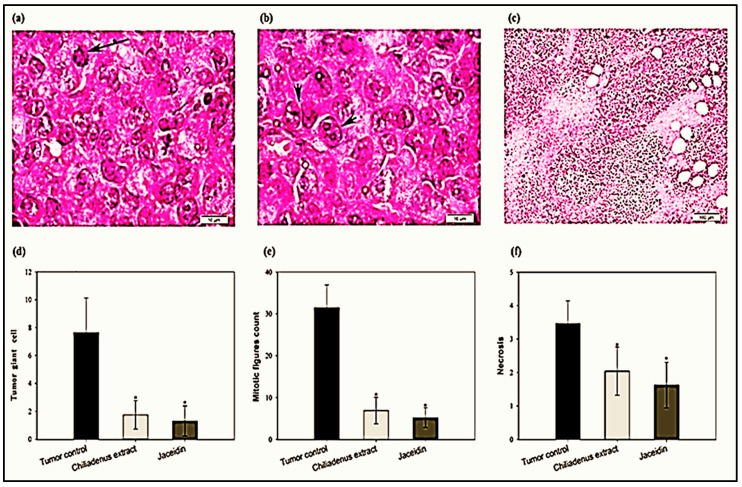
Effect of *Chiliadenus montanus* extract and jaceidin on tumor giant cell count (**a**,**d**); mitotic picture (**b**,**e**) and necrotic area (**c**,**f**) on Ehrlich’s carcinoma solid tumors growing in female mice. Data are expressed as the mean ± SD and analyzed using one-way ANOVA followed by Bonferroni’s post-hoc test. * compared to the tumor control group at *p* < 0.001.

The histological assay was performed by H&E staining to determine the number of giant cell (Figure 3a), mitotic picture (Figure 3b) and the extension of necrotic area (Figure 3c). The number of tumor giant cells in the tumor tissues was decreased in the experimental groups treated with *C. montanus* extract and jaceidin compared to tumor control groups (1.75 ± 1.02 and 1.30 ± 1.08 vs. 7.65 ± 2.48) at *p* < 0.001 (Figure 3d). Tumor giant cell was decreased significantly by treatment with *C. montanus* extract and jaceidin by 77.12% and 83 % respectively compared to tumor control.

Treatment with *C. montanus* extract or jaceidin reduced significantly the number of mitotic figures in the tumor tissues compared to tumor control groups (6.9 ± 3.16 and 5.20 ± 2.35 vs. 31.45 ± 5.46) at *p* < 0.001 (Figure 3e). Mitotic figure was decreased by treatment with *C. montanus* extract and Jaceidin by 78.06% and 83.47 % respectively compared to tumor control.

Necrosis in the tumor tissues was decreased in the experimental groups treated with *C. montanus* extract and Jaceidin compared to tumor control groups (2.04 ± 0.72 and 1.63 ± 0.68 vs. 3.46 ± 0.68 at *p* < 0.001 (Figure 3f). Tumor giant cell was decreased significantly by treatment with *C. montanus* extract and Jaceidin by 40.96% and 53.01 % respectively compared to tumor control.

Interestingly, the histopathological examination demonstrated several morphological changes in the experimental groups treated with *C. montanus* extract and Jaceidin compared to tumor control including irregularity of cell membrane, shrinkage and increased the number of apoptotic bodies and fragmentation of nuclei. These findings denote the ability of both *C. montanus* extract and Jaceidin in cell destruction and apoptosis and this is correlated with the reduction in tumor size.

#### 2.2.3. Enzyme-Linked Immunosorbent Assays

##### Determination of GSH and MDA Levels

**Figure 4:**
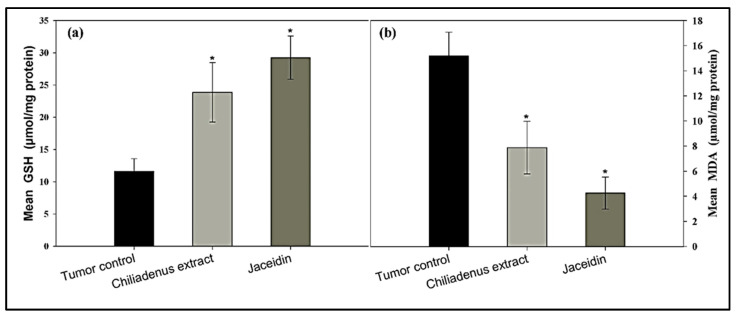
Effect of *Chiliadenus montanus* extract and jaceidin on GSH level (**a**) and MDA level (**b**) in Ehrlich’s carcinoma solid tumors growing in female mice. Data are expressed as the mean ± SD and analyzed using one-way ANOVA followed by Bonferroni’s post-hoc test. * compared to the tumor control group at *p* < 0.001.

GSH levels in tumor tissues were increased in the experimental groups treated with *C. montanus* extract and jaceidin compared to the tumor control group (23.88 ± 4.62 and 29.25 ± 3.36 vs. 11.62 ± 1.96) at *p* < 0.001 (Figure 4a). GSH was increased significantly at *C. montanus* extract and Jaceidin by 2 and 2.5 folds respectively compared to tumor control.

MDA levels in the tumor tissues were decreased in the experimental groups treated with *C. montanus* extract and jaceidin compared to tumor control groups (7.88 ± 2.09 and 4.25 ± 1.28 vs. 15.18 ± 1.89) at *p* < 0.001 (Figure 4b).

Statistical analysis showed highly significant difference among *C. montanus* extract and jaceidin in comparison to tumor control. MDA was decreased at *C. montanus* extract and jaceidin by 48% and 72% respectively compared to tumor control.

##### Estimation of VEGF-B Levels

**Figure 5:**
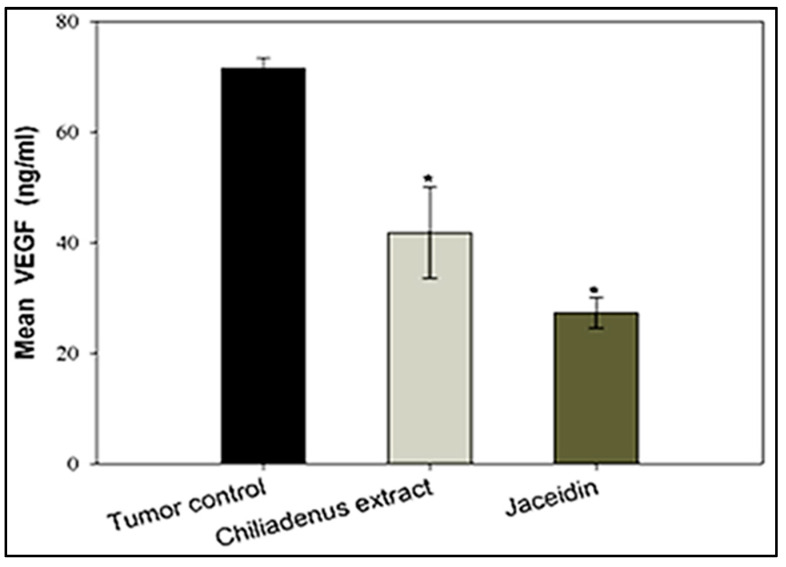
Effect of *Chiliadenus montanus* extract and jaceidin on serum VEGF level in female mice bearing Ehrlich’s carcinoma solid tumors. VEGF: vascular endothelial growth factor. Data are expressed as the mean ± SD and analyzed using one-way ANOVA followed by Bonferroni’s post-hoc test. * compared to the tumor control group at *p* < 0.001.

The serum levels of the vascular endothelial growth factor B (VEGF-B) was assessed by ELISA. The obtained results demonstrated that VEGF levels were lower in the experimental groups treated with *C. montanus* extract and jaceidin compared to tumor control group (41.78 ± 8.20 and 27.3 ± 2.82 vs. 71.46 ± 1.88) at *p* < 0.001 (Figure 5). Serum VEGF was decreased significantly at *C. montanus* extract and jaceidin by 41.54% and 61.7% respectively compared to tumor control.

Angiogenesis and vasculogenesis are essential processes in cancer progression, invasiveness and metastasis. They are regulated by multiple growth factors and cytokines mainly VEGF which is one of the most potent angiogenic factors involved in tumor growth and progression. VEGF induce endothelial cell proliferation, migration and tube formation by binding to tyrosine kinases (RTKs) receptors (VEGFR-1) and (VEGFR-2) expressed on endothelial cells.

Zajakowska and co-workers recognized that, the plasma level of VEGF-B was a sensitive marker in breast cancer [39]. It was found that overexpression of VEGF-B increased metastasis in pulmonary [40], bladder [41] and liver [42] cancers. Accordingly, targeting angiogenesis via selective inhibition of VEGFR kinase has been explored as a highly successful clinical strategy in cancer therapy. So far, several antiangiogenic drugs have been developed recently. It is noteworthy that some inhibitors such as bevacizumab, sunitinib and sorafenib have already used in clinical application [43,44]. The current study showed the ability of both *C. montanus* extract and Jaceidin to reduce VEGF suggesting their potential role as anti-angiogenic agents. This partly could explain the mechanism by which they reduce tumor size.

##### Molecular Modeling and Drug Design

**Figure 6:**
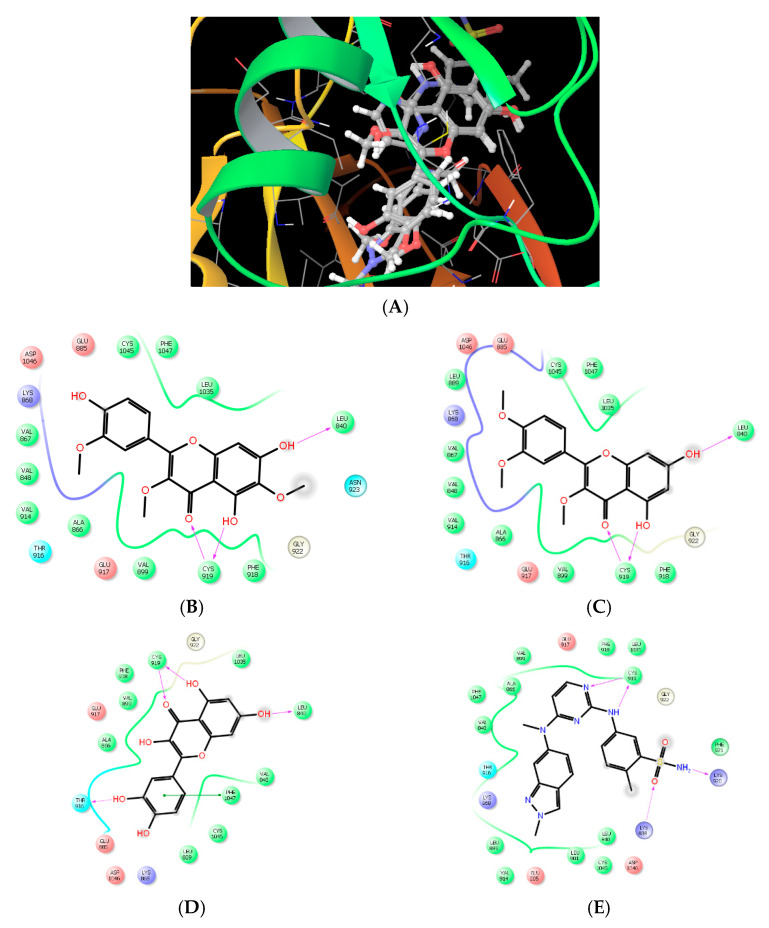
(**A**) 3D-Binding mode of VGEFR-ligand, compound 4, compound 3, quercetin, and genistein in the catalytic domain of 3EWH. 2D-ligand interaction diagram of compound 4 (**B**), compound 3 (**C**), quercetin (**D**), and pazopanib (**E**).

Nowadays, the coupling of computational, chemical, and biological techniques has extensively risen to streamline drug design and discovery. In the current study and in pursuance of predicting the anticancer mechanism of the isolated flavonoids, we have investigated the potential inhibitory effect of these compounds against VEGFR using molecular simulation with glide docking. Tyrosine kinase (TKs) proteins are divided into receptor and cellular (nonreceptor) associated domain, both of which are ATP-dependent proteins. ATP-binding pocket, a hydrophobic groove, is rich in alanine, valine, isoleucine, and leucine. This pocket is found in the hinge region linking the smaller N-terminal lobe to the larger C-terminal lobe of the catalytic domain. ATP-binding domain is conserved in TKs and serves as the receptor for anticancer agent. Beside the ATP-binding domain, there are other five potential pockets, which confer a degree of selectivity exhibited by various antineoplastic agents. Hydrogen-bonding, Van der Waals, hydrophobic, and electrostatic interactions play a vital role in holding ATP and tyrosine kinase inhibitors to the enzymatic domain. The discovery of new inhibitors has largely based on searching compounds containing the privilege chemicals moieties that can achieve the interaction requirement to adenine-binding site then optimize the compounds for selective binding to specific kinase. In the current study, the hydroxylated benzopyran ring is mimic to adenine; assuming so, the benzopyranone ketonic group (2.5 Å) and the nearby hydroxyl group (2.1 Å) form H-bond with the amide group of CYS919 in the hinge region (NH and carbonyl oxygen, respectively). Additionally, 7-hydroxyl group of benzopyranone can form extra hydrogen bond (2.3 Å) with carbonyl oxygen of LEU 840. These interaction forces help providing the tight contact of the flavonoid with VEGFR. Hydrophobic interaction is another force potentiates drug-receptor interaction. Phenyl moieties and methyl group of the current flavonoids exhibited Van der Waal and hydrophobic interaction with hydrophobic amino acids, THR 916, GLY922, VAL867, LEU889, PHE918, and LEU1035 in the hydrophobic groove of hinge region (Figure 6).

The docking protocol was repeated several times to select the best docking pose with the lowest energy. The results of docking protocols were identical; meaning a high reproducibility of the glide docking methodology. Extra precision glide docking of the flavonoid with VEGFR showed a strong docking score of −9.02 and −8.96 kJ/mol, and a glide Emodel value of −69.35 and −72.88 kcal mol^−1^ for compound 3 and 4, respectively. The reliability of the designed docking protocol was assessed by evaluating the interaction of the most known flavonoid of angiogenesis inhibitory activity, quercetin and genistein, with VEGFR. The quercetin and genistein exhibited the same pose and same kind of interaction, H-bonding and hydrophobic interaction, with nearly the same amino acids (Figure 6) as the compounds of interest achieving promising docking scores of −8.96 and −8.83 kJ/mol, and a glide Emodel value of −72.67 and −67.38 kcal mol^−1^ to quercetin and genistein, respectively. Under identical docking experimental conditions, pazopanib, a potent angiogenesis inhibitor approved by FDA in 2009, occupies the hydrophobic pocket of VEGFR exhibiting H-bonding, hydrophobic interactions. Pazopanib shows extra H-bond binding to VEGFR giving it the superiority of docking score (−9.5 kJ/mol) and glide Emodel (−93.65 kcal mol^−1^) (Figure 6).

##### In Silico ADME Predictive Study

The prediction of the drug-likeness of the current compounds as a potent angiogenesis inhibitor was assessed by studying their ADME (absorption, distribution, metabolism and excretion) properties using QikProp program v4.3 of the Schrodinger. This function provides an assessment of the physicochemical characters and the bioavailability of the extracted compounds based on their chemical structures. Many pharmacokinetic parameters were also investigated as presented in Table 2 such as polar surface area (PSA), QPPCaco (predicted apparent Caco-2 cell permeability in nm s^−1^), QPPMDCK (predicted apparent MDCK cell permeability in nm s^−1^).

Moreover, QPlogS (predicted aqueous solubility), QPlogKhsa (prediction of binding to human serum albumin), and percent human oral absorption (predicted human oral absorption on 0–100% scale) were also calculated. It is well known that an orally active compound should not have more than two violations of the Lipinski rule. The extracted flavonoids and reference flavonoid (Quercetin, Genistein) in this study were not violating the rule more than the maximum permissible limits and consequently confirming their drug-likeness properties. The molecular weight of the extracted flavonoids are less than 500 kDa and the number of hydrogen bond donor is less than 5 and less than 10 for hydrogen bond acceptor which play an important role in drug receptor interaction as shown under molecular modeling study. The oral bioavailability of the drug molecules could be controlled by the value of rotatable bonds (0–15) and polar surface area (PSA) (7–200 Å). The flavonoid showed a reasonable rotatable bond and polar surface area compared to quercetin and genistein. Compounds 3 and 4 have more or less the same rotatable of quercetin and have a lower polar surface area than quercetin and genistein, a property gave our compounds certain superiority regarding absorptivity. Another important property is the intestinal absorption or permeation; Caco-2 cell permeability (QPPCaco) is the good model to anticipate the intestinal permeation by passive diffusion transport. The extracted flavonoids exhibited the highest permeability, which confirmed by in vivo study in animals. Compound 3 is 25-fold more permeable than quercetin given the extract flavonoid the highest bioavailability compared to quercetin and genistein. On another hand, the blood brain barrier (BBB) permeability was expected by QPPMDCK descriptor. MDCK cells are an excellent model mimic to BBB. The extracted flavonoid showed a good BBB permeability (QPPMDCK > 25) compared to reference flavonoid. QPlogHERG, is another essential descriptor, which predicted IC_50_ value for blockage of HERG K^+^ channels. The flavonoids of interest are in the acceptable range of above −5.0, while the reference flavonoids are out. The long duration of action is a good character of the approved drug. Decreasing the dose frequency is an excellent property especially to cancer patient. The extract flavonoid could exhibit high binding to plasma albumin, which means longer duration of action. On another hand QPlogS and QPlogPo/w are descriptors of aqueous solubility and octanol/water partition coefficient; the extract compounds were found to be in the permissible range (Table 2). Briefly, our extracted compounds were considered promising lead molecules for designing potent and selective VEGFR inhibitors with excellent membrane permeability and oral bioavailability.

The docking and in silico results reveal that the phenolic compounds are powerfully interacting with the main amino acids residues of catalytic domain of VEGFR and have reasonable physicochemical and pharmacokinetic parameters making them promising lead compounds for the development of potent anti-cancer agent. Structural modification of the extracted flavonoids by addition of extra hydrogen bond donors and/or acceptor on the phenyl ring could enforce their binding to neighboring sites close to catalytic domains. By this tactic, we can develop a series of very potent drug-like candidates acting as angiogenesis inhibitors.

## 3. Material and Methods

### 3.1. Instruments

Instrumentation included JEOL spectrometer with tetramethylsilane as an internal standard for ^1^H (500 MHz) and ^13^C (125 MHz) NMR spectra. Materials used in chromatography; normal-phase silica gel for column chromatography (Fluka^®^, St. Louis, Mo, USA, 230–400 mesh), pre-coated TLC-plates ALUGRAM Xtra SIL G/UV254 (MACHEREY-NAGEL^®^, Düren, Germany, 0.2 mm) (normal phase), Sil G-25 unmodified standarad silica layers on glass for Preparative TLC, layer thickness 2 mm (MACHEREY-NAGEL^®^, Düren, Germany) and Sephadex LH-20 (Sigma Aldrich^®^, Darmstadt, Germany). Anisaldehyde–sulfuric acid was used as a spraying reagent.

### 3.2. Collection of Plant Materials

Aerial parts of *Chiliadenus montanus* (1.5 kg) were collected in June 2016 from Saint Katherine, South Sinai, Egypt. The plant was authenticated by Prof. Dr. Abdel Hamide Khedr, Department of Botany, Faculty of Science, Damietta University, Egypt. A voucher specimen (registration no: Cm-2016) was deposited the Herbarium of Pharmacognosy Department, Faculty of Pharmacy, Suez Canal University, Ismailia, Egypt. The collected plant was dried at room temperature then powdered.

### 3.3. Extraction and Isolation

*Chiliadenus montanus* powder (1 kg) was extracted twice by maceration with 100% chloroform (10 L, 3 days) at room temperature. The extract was concentrated in vacuo to give a residue (150 g). Only 20 g of the extract was fractionated on a silica gel column (90 × 5 cm) eluted with n-hexane-dichloromethane gradient until 100% dichloromethane. The *n*-hexane: CH_2_Cl_2_ (80:20) fraction (0.5 g) was chromatographed on silica gel cc (50 × 2 cm) using gradient system of n-hexane-ethyl acetate up to *n*-hexane-ethyl acetate (1:1) to afford 1 (5 mg) and 2 (6 mg). The *n*-hexane:CH_2_Cl_2_ (70:30) fraction (0.2 g) was purified by PTLC (*n*-hexane-CH_2_Cl_2_) (75:25) to afford 3 (30 mg). The *n*-hexane:CH_2_Cl_2_ (55:45) fraction (0.9 g) was applied to another silica gel column (50 × 2 cm) using *n*-hexane-ethyl acetate gradient (from 9:1 to 1:1) to yield sub fractions [A1–A5]. Sub fraction A1 showed promising TLC pattern, so Sub-fraction (A1) was re-purified using sephadex LH-20 column eluted with (*n*-hexane:CH_2_Cl_2_:methanol) (50:50:2.5) to give 4 (100 mg). The *n*-hexane: CH_2_Cl_2_ (40:60) fraction (0.4 g) was subjected to silica gel column separation (50 × 2 cm) using a gradient elution technique using *n*-hexane–ethyl acetate started with 40% ethyl acetate and culminated with 100% ethyl acetate to yield sub fractions [B1–B7]. B3 showed promising TLC pattern, so B3 sub fraction was re-purified using PTLC using (*n*-hexane:Ethyl acetate 45:55) as a developer to afford 5 (5 mg). The CH_2_Cl_2_:Methanol (92:8) (0.3 g) fraction was subjected to silica gel column separation (50 × 2 cm) eluted initially with 80% ethyl acetate–hexane up to 25% MeOH-ethyl acetate to yield sub fractions [C1–C4]. C3 showed promising TLC pattern, so Sub-fraction C3 was further purified by PTLC using 95% Ethyl acetate in Hexane as a mobile phase to afford 6 (7 mg).

The isolated compounds 1–6 (Figure 1) were identified by extensive study of their NMR spectral data as well as by comparison with the published data. Thus, the compounds were identified as Intermedeol (1) [45], 5α-hydroperoxy-β-eudesmol (2) [46], 5,7-dihydroxy-3,3’,4’-trimethoxyflavone (3) [11], 5,7,4’-trihydroxy-3,6,3’-trimethoxyflavone (jaceidin) (4) [47], Eudesm-11,13-ene-1β,4β,7α-triol (5) [15] and 1β,4β,7β, 11-tetrahydroxyeudesmane (6) [48].

### 3.4. Screening for Antitumor Activity

#### 3.4.1. In Vitro Assay

The cytotoxic activity of the isolated compounds was measured in vitro against a human liver cancer cell line (HEPG2), a human breast cancer cell line (MCF-7) and a human melanocyte (normal cell line) (HFB-4) in comparison to Doxorubicin, using sulforhodamine B (SRB) assay [49]. Human tumor cell lines (MCF-7, HepG2) used in this study were obtained from the American Type Culture collection (ATCC, Manassas, VA, USA). The tumor cell lines were maintained at the National Cancer Institute, Cairo, Egypt, by serial sub culturing. Cells were seeded in 96-well microliter plates at initial concentration of (3 × 103 cell/well) in a fresh medium and left for 24 h to attach to the plates before treatment of the tested compounds. Tested compounds were dissolved in DMSO and diluted with saline to the appropriate volume. After 24 h, cells were incubated with the appropriate concentration ranges of drugs (0, 12.5, 25, 50, 100 µg/mL), the wells were diluted to 150 µL with fresh medium and incubation was continued for 48 h at 37 °C. Control cells were treated with vehicle alone. Three wells were used for each drug concentration. After 48 h incubation, the cells were fixed with 50 µL of cold 10% trichloroacetic acid for 1 h at 4 °C, washed 5 times with distilled water using (Automatic Washer Tecan, Heidelberg, Germany) and then stained for 30 min at room temperature with 50 µL 0.4% SRB dissolved in 1% acetic acid. The wells were then washed 4 times with 1% acetic acid. The plates were air dried and the dye was solubilized with 100 µL/well 10 mM tris base (pH = 10.5). The Optical density (O.D.) of each well was measured spectrophotometrically at 570 nm with an ELISA microplate reader (sunrise Tecan reader, Germany). The percentage of cell survival was calculated as follows:

(1) Surviving fraction=O.D.(treated cells)/O.D.(control cells)

The relation between surviving fraction and compound concentration was plotted to get the survival curve for tumor cell line after the specified time. The concentration required for 50% inhibition of cell viability (IC_50_) was calculated for each tested compound (Table 1).

#### 3.4.2. In Vivo Assay

##### Induction of EAC Solid Tumors in Mice

Female Swiss albino mice with original body weight of 20–26 g and age of 8–10 weeks was used in the current experiment and fixed housing conditions were maintained at a normal dark/light cycle. Mice were housed in groups of six in polyethylene cages where food and water were provided ad libitum. The Research Ethics Committee at the Faculty of Pharmacy, Suez Canal University (license number 201809RA1), approved the study of protocol. Ehrlich’s Ascites Carcinoma is commonly employed as a solid form and easy to grow in suspension, when injected in the peritoneal cavity of female mice. Mice carrying the EAC cell line were obtained from the Department of Tumor Biology at the National Cancer Institute (Cairo, Egypt). The viability of EAC cells was ensured employing Trypan blue dye exclusion method. Then, EAC cells suspension was prepared in sterile saline solution to get a final working suspension; each 0.1 mL of which contained 2.5 million of EAC cells. At the beginning of the experiment, mice were shaved at their back, and inoculated with 0.1 mL of the EAC suspension.

##### Experimental Design

Seven days after inoculation with the tumor cells in all female mice, mice were randomly divided into three groups, ten mice in each. The different treatments were started as follows;

Groups 1: mice treated with saline (5 mL/kg). All therapies continued for 21 days. Group 2: mice treated with *C. montanus* extract 200 mg/kg three times weekly. Group 3: mice treated with Jaceidin 50 mg/kg three times weekly.

One day after the end of the experiment (day 21), mice were subjected to light ether anesthesia and sacrificed by cervical dislocation; blood samples were collected by cardiac puncture. Blood samples were maintained at room temperature for 30 min and centrifuged at 12,000× *g* for 10 min. Then, sera were separated and stored at −20 °C until used. An ELISA kit (Sun Red Biotechnology Company, Shanghai, China) was employed for estimating serum VEGF level according to the manufacturer’s protocol. The color intensity was measured at 450 nm using an automated ELISA reader (Europe S.A., Belgium). Tumor was dissected into two halves; the first half was kept at −20 °C to be used in estimating MDA and GSH levels. The second half was cut into small pieces, then fixed in 10% phosphate-buffered formalin for histopathological examination.

##### Histopathological Examination of Solid Tumors

After fixation, samples were dehydrated in ascending grades of ethyl alcohol, cleared in xylol, embedded in paraffin wax, and then subjected to routine histopathological procedures. Sectioning was done at 5–7 µm thick followed by staining with Hematoxylin and Eosin (H&E). Digital imaging of representative areas was captured using image capture engine software (AMT V600.259) which attached to Olympus CX 41biocular microscope. Histopathological analysis of the digital images of solid tumor was performed by quantities the mitotic figures and tumor giant cells. For counting, ten sections from each experimental group were analyzed at scale bar 20 µm by using image J software that was developed by the National Institute of Health (Betheda, MD, USA). Histopathological examinations were evaluated blindly.

#### 3.4.3. Enzyme-Linked Immunosorbent Assays

Using a frozen sample, a part of tumor was weighted and homogenized as 10% (w/v) in phosphate-buffered saline using a Teflon homogenizer (Glass col homogenizer system, Vernon Hills, IL, USA). Homogenates were then centrifuged at 15,000× *g* for 10 min at 4 °C. After centrifugation, the supernatants were collected into 2 dry tubes and kept at −80 °C. The levels of MDA and GSH were determined in the tumor homogenates of rats using ELISA kits for MDA (Catalog No. MBS741034) and GSH (Catalogue Number: MBS026635) according to the manufacturer’s instructions.

#### 3.4.4. Molecular Modeling and Drug Design

In the current research, a molecular modeling study was conducted using the Glide docking function incorporated in the Schrodinger-10.1 molecular modeling program. The X-ray crystal structure of the catalytic domain of VEGFR in complex with Pyridyl-pyrimidine benzimidazole (PDB ID: 3EWH resolution 2.6 Å) was obtained from Protein Data Bank (PDB) The VEGFR– Pyridyl-pyrimidine benzimidazole complex was refined for the Glide docking calculations using the protein preparation wizard applying the OPLS-2005 force field. In the second step, crystallographic water, if present, was removed, and hydrogens were added to the structure corresponding to pH 7.0, most likely positions of hydroxyl and thiol hydrogen atoms, considering the appropriate ionization states for both the basic and acidic amino acid residues of the protein. In the third step, the appropriate charge and protonation state of protein were adjusted by the protein assignment script, then the protein-inhibitor complex was subjected to energy minimization until the average root mean square deviation (RMSD) of the non-hydrogen atoms reached 0.3 Å in order to release the steric clashes using the OPLS-2005 force field [28].Using ligand preparation wizard, the 3D structures of the extracted flavonoids were constructed and optimized with the build panel in Maestro. The ligand preparation function generates several low energy 3D structures with various ionization states, tautomers, stereochemistries, and ring conformations, for each input molecule. Partial atomic charges were ascribed for flavonoid derivatives using the OPLS-2005 force-field, and possible ionization states were generated at a pH of 7. To soften the potential for non-polar parts of the receptor, the van der Waal radii of receptor atoms were scaled by 0.8 with a partial atomic charge of 0.15. A grid box with coordinates X = 10, Y = 10 and Z = 10 was generated at the centroid of the active site. The ligand structures thus obtained were further optimized by energy minimization until it reached RMSD cutoff of 0.01 Å. The properties and the shape and of the active site of VEGFR were characterized using “grid generation panel” in Glide after ensuring that the VEGFR receptor and phenolic molecules were in the correct form.

In the final step, the flavonoids were docked within the active site of VEGFR using the optimized protein-ligand geometries. The extra precision (XP) Glide scoring function, which docks ligands flexibly, is applied to rank the docking poses and to assess the protein-ligand binding affinities. Maestro’s Pose Viewer utility was utilized to visualize and analyze the key elements of ligand- receptor interaction. The final best-docked structure with the lowest-energy was chosen using a glide score function and selected for further experiments. Erlotinib was removed from the crystal structure VEGFR receptor then re-docked using the previous-mentioned step to evaluate the accuracy and precision of established docking protocol.

#### 3.4.5. In Silico ADME Predictive Study

Drug-likeness properties of natural compounds, with expected biological and/or pharmacological activity, were evaluated according Lipinski’s rule of five, which utilized to determine whether these compounds have the properties that would allow them a likely orally active drug in humans. The Lipinski’s rule of five prescribe important pharmacokinetics properties in the human body, including its absorption, distribution, metabolism, and excretion (ADME). The drug-like behavior of our compounds was predicted by using QikProp module (v4.2, Schrodinger 2015-1). The flavonoids prepared by LigPrep module (v3.1, Schrodinger 2015-1) in the previous step were applied for the calculation of pharmacokinetic parameters by QikProp v4.2, which utilizes the method of Jorgensen19 to compute pharmacokinetic properties and descriptors physically significant parameters and pharmaceutically relevant properties and of all the synthesized compounds such as molecular weight, log p, H-bond donors, and H-bond acceptors were analyzed [50].

#### 3.4.6. Statistical Analysis

Data were collected, tabulated and presented as the mean ± standard error of the mean (S.E.M.) One-way ANOVA followed by Bonferroni’s post-hoc test were used to analyze the difference between the experimental groups. All statistical tests were performed using SPSS 22. A *p*-value < 0.05 was considered to be statistically significant.

## 4. Conclusions

In the current study, phytochemical investigation of *C. montanus* has led to the isolation of two methoxy flavonoids; one of them is jaceidin, along with four sesquiterpenes. Jaceidin exhibited potent in vitro cytotoxicity and displayed promising in vivo antitumor effect. Jaceidin suppressed the progression of Ehrlich’s ascites carcinoma in mice by decreasing the serum levels of VEGF and alleviating the oxidative stress via increasing GSH levels and decreasing MDA levels. In order to confirm the angiogenesis inhibition activity of jaceidin, the binding affinity of the isolated flavonoids towards VEGFR was determined by molecular simulation using glide docking. The docking results exhibited that three binding forces including, hydrogen bonds, hydrophobic, and hydrophilic interactions were participated in the tight binding of the phenolic compounds to VGEFR. Consequently, the activity of VEGFR mediated signaling cascades was suppressed resulting in the inhibition of angiogenesis. These findings suggested that the isolated flavonoids—and jaceidin in particular—are promising lead molecules for designing potent and selective VEGFR inhibitors with excellent membrane permeability and oral bioavailability.

## Figures and Tables

**Table 1:** Cytotoxic activity of the tested compounds against liver cancer cell line (HepG2), breast cancer cell line (MCF-7) and a human melanocyte (normal cell line) (HFB-4) in vitro.

Compound No.	HepG2 (IC_50_) ^a,b^ in µM	MCF-7 (IC_50_) ^a,b^ in µM	HFB-4 (IC_50_) ^a,b^ in µM
1	48.2 ± 0.30	45.6 ± 0.50	>200
2	39.7 ± 0.65	41.5 ± 0.55	>200
3	30.5 ± 0.61	10.7 ± 0.40	>200
4	9.7 ± 0.26	9.3 ± 0.30	>200
5	33.4 ± 0.49	38.5 ± 0.55	>200
6	35.1 ± 0.60	33.2 ± 0.35	>200
Doxorubicin ^c^	4.58 ± 0.47	4 ± 0.25	14.7 ± 0.50

^a^ Dose of the compound which inhibit tumor cell proliferation by 50%. ^b^ Each experiment was carried out in triplicate and the results were expressed as ±SD. ^c^ Used as positive control.

**Table 2:** In silico absorption, distribution, metabolism and excretion (ADME) prediction parameters of isolated and reference molecules.

ADME Prediction Parameters	Compound		Reference Flavonoid	
	3	4	Quercetin	Genistein
Mol-MW ^a^	344.3	360.3	302.2	286.2
DonorHB ^b^	1	2	3	4
AcceptHB ^c^	5.2	6	5.2	4.5
QPlogPo/w ^d^	2.7	2.2	0.517	1.05
#rotor ^e^	5	6	5	4
PSA ^f^	95.3	115.5	140.08	118.6
QPlogS ^g^	−4.1	−3.8	−3.09	−3.05
QPlogHERG ^h^	−4.9	−4.7	−5.3	−5.07
QPPCaco ^i^	549.5	287.7	21.03	58.2
QPP MDCK ^j^	259.01	128.7	7.62	22.89
QPlogKhsa ^k^	0.163	0.013	−0.318	−0.198
% Human Oral Absorption ^l^	92.2	83.92	53.65	64.7

Acceptable Ranges: ^a^ <500 amu; ^b^ <5; ^c^ <10; ^d^ <5; ^e^ 0–15; ^f^ 7–200; ^g^ <0.5; ^h^ <−5; ^i^ <25 poor, >500 great; ^j^ <25 poor, >500 great; ^k^ −1.5–1.5; ^l^ >80% is high, <25% is poor.
